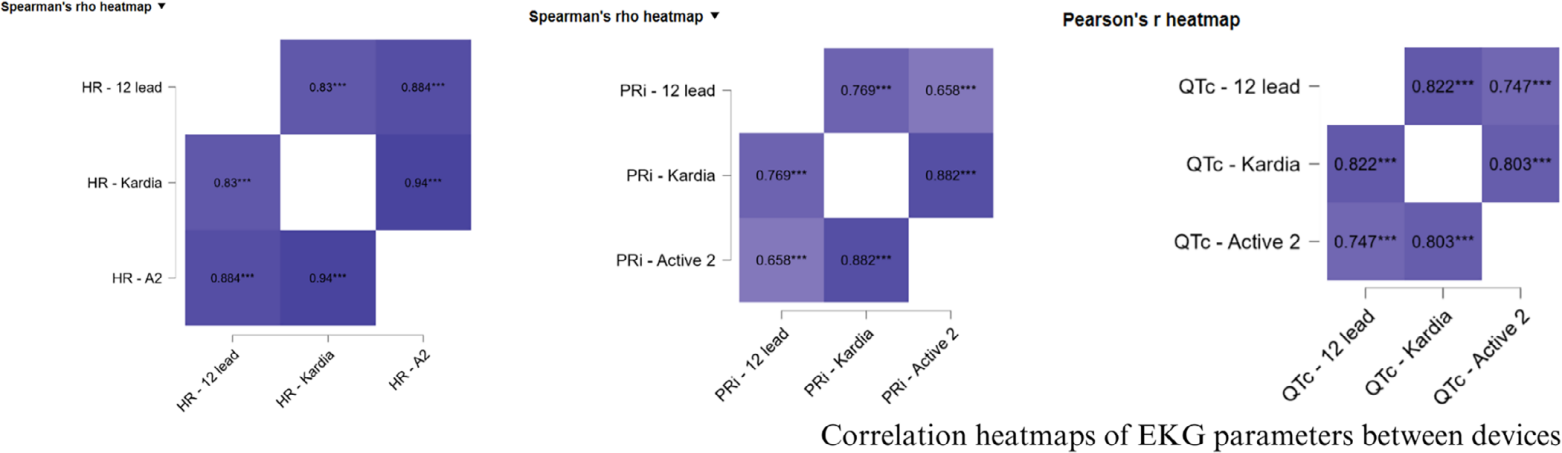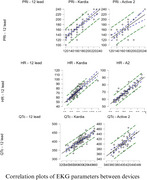# Using portable electrocardiogram devices to assess cardiovascular issues related to pharmacotherapy in Alzheimer's disease patients

**DOI:** 10.1002/alz70858_100005

**Published:** 2025-12-25

**Authors:** Leonardo Ryuiti Kimoto, Júlia de Almeida Barreto, João Vitor da Silva Viana, Julia Cardoso Costa Cardoso Costa, Giovanna Correia Pereira Moro, Bárbara Lopes Farace, Joice Coutinho de Alvarenga, Gabriela Tomé Oliveira Engelmann, Marco Aurélio Romano‐Silva, Jonas Jardim de Paula, Maria Aparecida Camargos Bicalho, Bernardo de Mattos Viana, Cog‐Aging Research Group

**Affiliations:** ^1^ Cog‐Aging Research Group, Belo Horizonte, Minas Gerais, Brazil; ^2^ Undergraduate Medicine, Faculty of Medicine, Universidade Federal de Minas Gerais (UFMG), Belo Horizonte, Minas Gerais, Brazil; ^3^ Neurotec R National Institute of Science and Technology (INCT‐Neurotec R), Faculty of Medicine, Universidade Federal de Minas Gerais (UFMG), Belo Horizonte, Minas Gerais, Brazil; ^4^ Cog‐Aging Research Group, Universidade Federal de Minas Gerais (UFMG), Belo Horizonte, Minas Gerais, Brazil; ^5^ Older Adult's Psychiatry and Psychology Extension Program (PROEPSI), School of Medicine, Universidade Federal de Minas Gerais (UFMG), Belo Horizonte, Minas Gerais, Brazil; ^6^ Older Adult's Psychiatry and Psychology Extension Program (PROEPSI), Belo Horizonte, Minas Gerais, Brazil; ^7^ Molecular Medicine Postgraduate Program, School of Medicine, Universidade Federal de Minas Gerais (UFMG), Belo Horizonte, Minas Gerais, Brazil; ^8^ Universidade Federal de Minas Gerais, Belo Horizonte, Brazil; ^9^ Jenny de Andrade Faria Institute – Outpatient Reference Center for the Elderly, Universidade Federal de Minas Gerais (UFMG), Belo Horizonte, Minas Gerais, Brazil; ^10^ Molecular Medicine Program, School of Medicine, Federal University of Minas Gerais, Belo Horizonte, Minas Gerais, Brazil; ^11^ Department of Psychiatry, School of Medicine, Federal University of Minas Gerais, Belo Horizonte, Minas Gerais, Brazil; ^12^ Universidade Federal de Minas Gerais, Belo Horizonte, Minas Gerais, Brazil; ^13^ INCT – NeuroTecR and CTMM, Belo Horizonte, Minas Gerais, Brazil; ^14^ Older Adult's Psychiatry and Psychology Extension Program I Federal University of Minas Gerais, Belo Horizonte, MG, Brazil; ^15^ Graduate Program in Applied Sciences to Adult Health, Faculdade de Medicina, Universidade Federal de Minas Gerais, Belo Horizonte, Brazil; ^16^ Graduate Program in Molecular Medicine, Belo Horizonte, Minas Gerais, Brazil; ^17^ UFMG, Belo Horizonte, Brazil; ^18^ Geriatrics and Gerontology Center Clinical Hospital of University of Minas Gerais, Belo Horizonte, Minas Gerais, Brazil; ^19^ Department of Internal Medicine, School of Medicine, Federal University of Minas gerais, Belo Horizonte, Minas Gerais, Brazil; ^20^ Sciences Applied to Adult Health Postgraduate Program, School of Medicine, Universidade Federal de Minas Gerais (UFMG), Belo Horizonte, Minas Gerais, Brazil; ^21^ Federal University of Minas Gerais, Belo Horizonte, Minas Gerais, Brazil; ^22^ National Institute of Science and Technology (INCT‐Neurotec R), Faculty of Medicine, Federal University of Minas Gerais, Belo Horizonte, Minas Gerais, Brazil; ^23^ Older Adult's Psychiatry and Psychology Extension Program Federal University of Minas Gerais, Belo Horizonte, Minas Gerais, Brazil; ^24^ Jenny de Andrade Faria Institute – Outpatient Reference Center for the Elderly, Universidade Federal de Minas Gerais (UFMG), Belo Horizonte, Minas Gerais, Brazil; ^25^ Hospital das Clínicas da UFMG, University Hospital, Universidade Federal de Minas Gerais (UFMG), Belo Horizonte, Minas Gerais, Brazil

## Abstract

**Background:**

It is predicted that the prevalence of Alzheimer's disease (AD) in low and middle income countries (LMIC) will rise more rapidly than in high‐income countries. Pharmacotherapy for AD are associated with a variety of cardiovascular (CV) issues including heart block, sinus bradycardia and syncope. Regarding this fact, it is of utmost importance to evaluate CV health before starting therapy, with EKG being an important tool to evaluate heart blocks, bradycardia, rhythm disturbances and prolonged QTc.

Recently multiple EKGs portable devices were released. They can potentially be used to help the decision making in AD therapy in a more accessible and faster way in remote outpatient settings in LMIC, where EKGs aren’t as easily available.

**Method:**

To compare the accuracy of 2 portable EKG devices, using a 12‐lead‐EKG as baseline, one hundred participants followed in Cog‐Aging cohort in Brazil were submitted to EKG evaluation. All were assessed by 12‐lead‐EKG, 95 of them by Kardiamobile 6L and 52 by Samsung Watch Active 2. Normality of distribution was assessed by Shapiro‐Wilk test, accuracy of QTc interval was assessed by Pearson's correlation test, and HR and PRi accuracy was evaluated by Spearman's correlation test.

**Result:**

The patients presented a median age of 77, with 76% being women, 51% white, 46% presenting MCI, 34% presenting probable AD dementia (ADD) and 20% with no cognitive impairment. Comparisons between the 3 devices did not show statistical differences for QTc (*p* = 0,412) in ANOVA test and for PRi (*p* = 0.383), HR (*p* = 0,159) in Kruskal‐Wallis test. Kardia L6 presented a stronger correlation with 12‐lead‐EKG for PRi(rho = 0.769) and QTc (*r* = 0,822) and Samsung's device presented a stronger correlation for HR(rho = 0.884)

**Conclusion:**

This is a preliminary study regarding the use of portable EKG devices to assess cardiovascular issues related to pharmacotherapy in AD. Considering its ease of use, accessibility and accuracy, portable EKG are a promising technology to make decision‐making faster and more immediate in remote outpatient settings.